# Feasibility and Efficacy of S-Adenosyl-L-methionine in Patients with HBV-Related HCC with Different BCLC Stages

**DOI:** 10.1155/2016/4134053

**Published:** 2016-11-28

**Authors:** Tao Guo, Yukun He, Weijie Ma, Zhisu Liu, Quanyan Liu

**Affiliations:** Department of General Surgery, Research Center of Digestive Diseases, Zhongnan Hospital of Wuhan University, Wuhan 430071, China

## Abstract

*Aims*. To understand the feasibility and efficacy of treatment with SAMe in patients with hepatitis B-related HCC with different Barcelona Clinic Liver Cancer (BCLC) stages.* Methods*. We retrospectively enrolled 697 patients with BCLC early-stage (stages 0*-*A) and advanced-stage (stages B*-*C) HCC who underwent SAMe therapy (354 cases) or no SAMe therapy (343 cases). The baseline characteristics, postoperative recoveries, and 24-month overall survival rates of the patients in the 2 groups were compared. Cox regression model analysis was performed to confirm the independent variables influencing the survival rate.* Results*. For patients in the early-stage (BCLC stages* A1–A4*) group, little benefit of SAMe therapy was observed. For advanced-stage (BCLC* B-C*) patients, SAMe therapy reduced alanine aminotransferase (ALT) and aspartate transaminase (AST) levels and effectively delayed the recurrence time and enhanced the 24-month survival rate. Cox regression model analysis in the advanced-stage group revealed that treatment with SAMe, preoperative viral load, and Child-Pugh grade were independent variables influencing survival time.* Conclusion*. SAMe therapy exhibited protective and therapeutic efficacy for BCLC advanced-stage HBV-related HCC patients. And the efficacy of SAMe therapy should be further explored in randomized prospective clinical trials.

## 1. Introduction

Hepatocellular carcinoma (HCC) is among the most common cancer types and causes of cancer-related mortality worldwide [1]. HCC nearly always develops within the background of chronic liver disease. Globally, nearly 50% of all HCC cases are attributable to chronic hepatitis B virus (HBV) infection [2]. Surgery remains the most effective treatment with curative potential. However, only approximately 10 to 20% of patients with HCC are eligible for surgical intervention. Therapies such as transcatheter arterial chemoembolization (TACE) and interferon alpha may prolong survival in some patients. However, the response to these treatments is often not satisfactory because of the high rate of relapse. Thus, novel drugs that can induce synergistic therapeutic effects in HBV-related HCC patients are urgently needed.

S-Adenosyl-L-methionine (SAMe) is synthesized by methionine adenosyltransferase and functions as the principal biological methyl donor in the liver [3, 4]. SAMe plays a critical role in the synthesis of polyamines and provides cysteine for the production of glutathione (GSH) [5]. Dysregulation of methionine metabolism has been implicated in human liver cancer. In hepatocytes, SAMe not only serves as a precursor for GSH synthesis but also positively regulates the expression of glutamate-cysteine ligase (GCL), the rate-limiting enzyme in GSH synthesis [6–8]. Based on these multiple activities, SAMe is employed as a hepatoprotective agent in chronic liver diseases and has curative effects for liver injury induced by carbon tetrachloride, acetaminophen, alcohol, and ischemia-reperfusion [9–12]. In addition, SAMe also has protective effects against intrahepatic cholestasis in pregnancy [13] and improves the survival rate of patients with alcoholic liver disease [14]. In summary, there is strong preclinical evidence that SAMe has important physiological roles in health and clinical efficacy in liver disease [15]. However, the clinical efficacy of SAMe for HBV-related HCC with different BCLC stages still remains uncertain.

A high viral load is an independent risk factor for recurrence of hepatitis B virus- (HBV-) related hepatocellular carcinoma (HCC) after surgery [16–18]. SAMe may improve interferon signaling and antiviral effects through increased methylation of STAT1, resulting in enhanced STAT1-DNA binding and thus greater ISG expression [19]. SAMe is the first interferon sensitizing agent with in vivo efficacy, suggesting its utility as an adjunct to interferon-based therapy; such applications may be particularly important in the era of direct antivirals for HBV infection. In addition, we have demonstrated that plasma SAMe levels are associated with the severity of HBV-related liver disease [20]. Thus, treatment of HBV-related HCC with SAMe may induce therapeutic effects by improving liver function and the effectiveness of the antiviral response. In this study, we retrospected former controlled clinical trial to evaluate the effectiveness and safety of SAMe therapy for HBV-related HCC with different BCLC stages (early stage and advanced stage).

## 2. Materials and Methods

### 2.1. Description of Study Design

**Figure 1:**
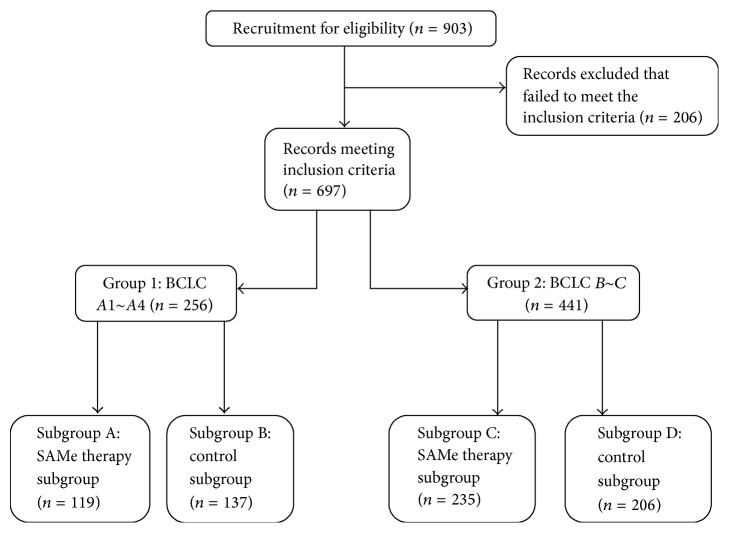
Flow diagram of the process for including patients in this study.

We retrospectively selected appropriate HCC patients from 2004 to 2013 (follow-up to early 2016) with chronic hepatitis B (CHB) for this research. Written informed consent was obtained from all subjects and submitted to the ethics committee normally. The protocol for this research was carried out in accordance with the approved guidelines and approved by Licensing Committee of Wuhan University. Since then, all experiments were performed in accordance with relevant guidelines and regulations. We defined early stage as BCLC stage 0 and stage A (A1~A4) and advanced stage as BCLC stage B, stage C, and stage D. All included patients were classified into 2 groups according to BCLC stage (early stage, group 1; advanced stage, group 2). In each group, the participants were assigned to 2 subgroups based on acceptance or nonacceptance of SAMe therapy. We discussed their conditions with each patient but the decision of SAMe therapy was made by their own. Relevant parameters were measured during the perioperative period for comparison between the SAMe therapy subgroup and control subgroup of each group. Postoperative long-term characteristics, including recurrence time and survival rate, were also analyzed between the subgroups. The flow of allocation of the recruited patients is presented (Figure 1). In addition, postoperative and long-term SAMe treatment were the only interventions in each group.

### 2.2. Patient Selection

The inclusion criteria for the recruited patients were as follows: (1) hepatitis B surface antigen- (HBsAg-) positive state for more than 6 months with cirrhosis; (2) elevated alanine transaminase (ALT) or total serum bilirubin (TBIL) levels or features of chronic hepatitis in liver pathology; (3) positive for hepatitis B e antigen (HBeAg), anti-HBe, or HBV-DNA; (4) negative for anti-HBcIgM titer 1 : 1000; and (5) confirmation of tumorous characteristics and description of hepatocellular carcinoma by postoperative pathology.

To establish these criteria, we first obtained all medical records for patients with HBV-related HCC. Next, we excluded patients who died during the perioperative period or did not undergo surgery. Patients with other severe complications (such as preoperative tumor rupture, severe hypoproteinemia, or Child-Pugh class C) or a history of previous liver surgery (such as intrahepatic duct calculus and giant hepatic cyst) were also excluded. Patients who received preoperative chemotherapy were also excluded, as were patients who underwent additional surgical procedures during hospitalization or with intraoperatively confirmed tumor metastasis. Among patients with large lesions (>5 cm), only those with single large lesions were included. Patients with several lesions confined to the same area were also included. In addition, at entry, none of the included patients received continuous antiviral therapy before. Decisions concerning the eligibility of individual patients were made in an unblinded manner.

### 2.3. Perioperative Period and Long-Term Information Collection

General information (such as gender, age, and hepatitis viral load) was collected using questionnaires and blood sample tests. Preoperative ALT, AST, TBIL, and ALB values were used to evaluate the hepatic injury response and hepatic functional reserve after surgery. These parameters were also measured on postoperative days 1, 3, and 7. Preoperative liver CT scans were performed to describe the size and number of lesions; the characteristics of the lesions and liver tissues were confirmed and recorded during the operations. The surgical time was recorded, and hemorrhage was assessed based on aspirated fluids. In addition, all postoperative information was confirmed by reviewing medical records, including for postoperative complications (such as postoperative peritoneal effusion or biliary fistula). For long-term information collection, the recurrence times of the included patients were recorded based on the results of postoperative continuous reexaminations. Death rates were calculated until the 24th month since first entry by telephone follow-up interviews. Patients lost to follow-up were not included in this study.

### 2.4. Treatment

In this study, the only surgical protocol was the traditional open procedure, which was performed by three experienced surgeons and their coworkers. Subcostal incision was performed for all included patients. For each patient, the characteristics of the lesions were carefully confirmed by intraoperative manual exploration and pathological anatomy. After exploration, intraoperative ultrasound was used to exclude previously undetected lesions or/and to localize tumors that are situated under the liver surface and are not palpable. Anatomical hepatic resection with en bloc thrombectomy was our preferred surgical method for liver resection. As an alternative, nonanatomical resection was used in cases of where wide en bloc resection was not feasible. The tumor thrombus was either resected en bloc with the tumor or extracted out of the vascular lumen, depending on its location and extent. Pringle's maneuver was routinely used with a clamp/unclamp time of 10/5 min. We completed the operations after searching the abdominal cavity to confirm the extent of local disease and extrahepatic metastases. No staging operations were performed in any of the included cases.

During the perioperative period (from 3 days prior to surgery to the 7th postoperative day), patients were assigned to receive magnesium isoglycyrrhizinate (MgiBC) (Chai Tai Tianqing Pharmaceutical Group Co., Ltd., China) injection 100 mg per day. S-Adenosyl-methionine (SAMe) was also administered to patients in the SAMe therapy subgroups (*subgroup A and subgroup C,* Figure 1) (Knoll Farmaceutici SPA, Via Europa, Italy) 1000 mg per day (slow intravenous drip, 500 mg, 2 times a day) during the perioperative period. The patients in subgroups A and C also received SAMe (Knoll Farmaceutici SPA, Via Europa, Italy) 1500 mg tablets daily (take orally, 500 mg, 3 times a day) for 24 months after leaving the hospital. Telephone reminders and regular periodic prescriptions were continued during the follow-up period. No specific postoperative treatment other than conventional treatments (i.e., acid suppression, anti-infection, and hemostasis) were provided to the patients during their hospital stay. Each patient with tumor recurrence was treated through combined therapy (such as absolute alcohol injecting or radiofrequency ablation) without secondary surgery. After leaving hospital, lifetime antiviral therapy was administered in the form of oral entecavir (Bristol-Myers Squibb Co., Ltd., USA) 500 mg per day.

### 2.5. Statistical Analysis

Data for continuous variables (including the recurrence time) are presented as the mean ± standard deviation (SD) and were compared using Student's *t*-tests. Some data (e.g., gender or postoperative complications) were collected as categorical variables. Pearson's chi-square test or Fisher's exact test was used to compare categorical data. Moreover, survival rates were collected and compared using the log-rank test for the analysis of prognosis. Multivariate analysis was performed based on two subgroups using the Cox regression model in case significant survival rates existed; HBV-related variables (such as preoperative viral road) were selected.

Differences were considered significant at *P* < 0.05. Statistical analyses were performed using SPSS software (233 South Wacker Drive, Chicago, USA, version 17.0), and figures were prepared using GraphPad Prism software (7825 Fay Avenue, La Jolla, USA, version 5.0).

## 3. Results

Of the 903 reviewed medical records, 697 patients met the inclusion criteria. No BCLC stage 0 or stage D patients were included in this study. We performed detailed statistical analyses of the general information and preoperative data. The general information for the 2 subgroups in each group were well matched at entry, and no significant differences were identified, including the preoperative parametric data (Table 1). In the follow-up interviews, none of the included patients dropped out of the prognosis analysis, and no liver transplantations were performed by the end point (Figure 1).

### 3.1. Analysis of Patients in the Early-Stage Group during the Perioperative Period and Prognosis

**Figure 2:**
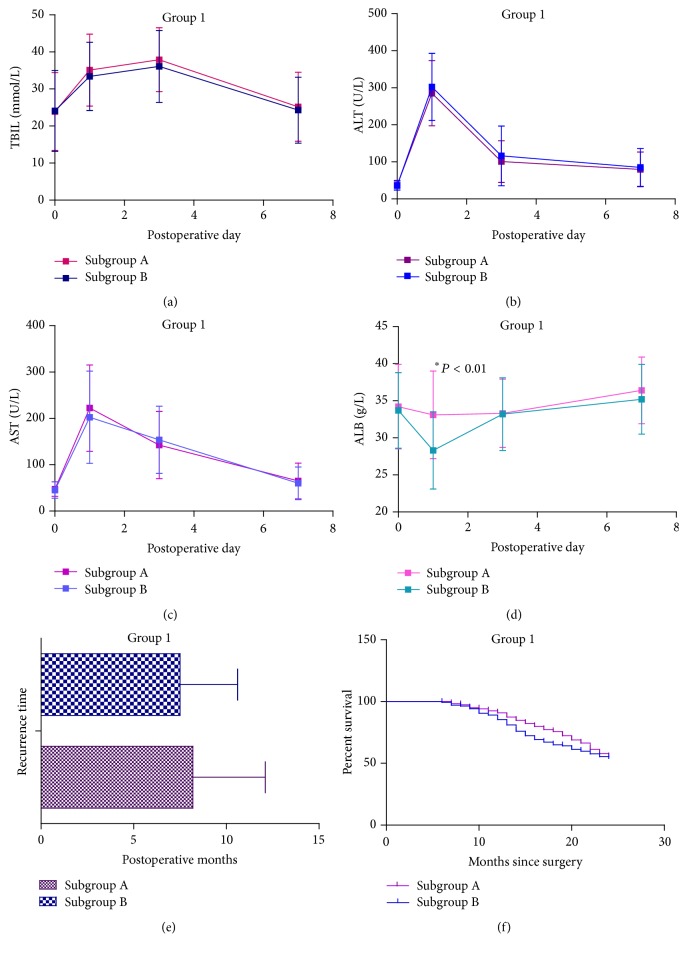
Curves of parametric data for patients in group 1 during the perioperative period and prognosis data. (a) Curves of TBIL levels in group 1 on postoperative days 1, 3, and 7. (b) Curves of ALT levels in group 1 on postoperative days 1, 3, and 7. (c) Curves of AST levels in group 1 on postoperative days 1, 3, and 7. (d) Curves of ALB levels in group 1 on postoperative days 1, 3, and 7. (e) Recurrence time of patients in group 1. (f) Cumulative survival of all included patients in group 1 in the first 24 postoperative months.

We compared parametric data, including TBIL, ALT, AST, and ALB levels, during the perioperative period between subgroup A and subgroup B in early-stage group, which comprised 256 patients (119 patients received SAMe therapy). ALB levels on operative day 1 were significantly higher in the SAMe therapy subgroup (subgroup A) than subgroup B (33.1 ± 5.9 versus 28.3 ± 5.2, *P* < 0.01) (Figure 2(d)). The remaining parametric data in group 1 did not differ significantly (Figure 2). Other postoperative data, including postoperative ICU stay, including postoperative complications, surgical time, and hospital stay, also did not differ significantly (Table 2).

In the analysis of long-term prognosis in the early-stage group, the recurrence time of patients in subgroup A (119 patients) did not differ significantly from that of subgroup B (137 patients) (Figure 2(e)). In addition, in the cumulative 24-month survival curve, log-rank tests revealed no significant difference between subgroups A and B (Figure 2(f)).

### 3.2. SAMe Has Significant Clinical Effects in Advanced-Stage Patients

**Figure 3:**
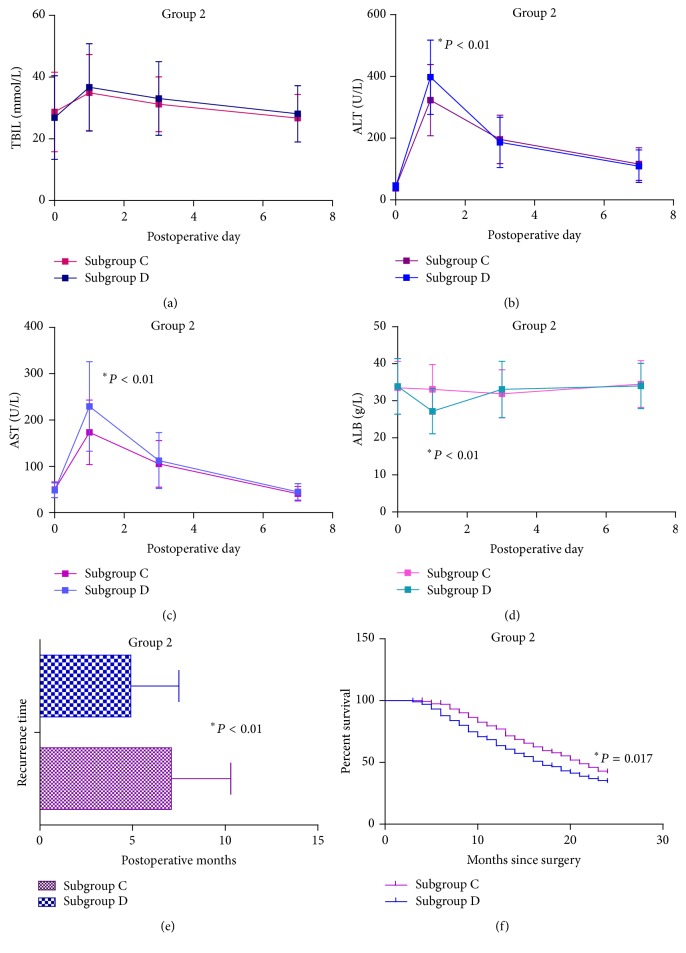
Curves of parametric data for patients in group 2 during the perioperative period and prognosis data. (a) Curves of TBIL levels in group 2 on postoperative days 1, 3, and 7. (b) Curves of ALT levels in group 2 on postoperative days 1, 3, and 7. (c) Curves of AST levels in group 2 on postoperative days 1, 3, and 7. (d) Curves of ALB levels in group 2 on postoperative days 1, 3, and 7. (e) Recurrence time of patients in group 2. (f) Cumulative survival of all included patients in group 2 in the first 24 postoperative months.

To analyze the efficacy of SAMe during the perioperative period in advanced-stage group, we compared relevant parametric data, including liver function on postoperative days 1, 3, and 7, postoperative complications, and hospital stay between subgroups C and D. ALT and AST levels on postoperative day 1 were significantly lower in subgroup C than subgroup D (323.1 ± 115.2 versus 397.5 ± 120.4, 173.5 ± 69.8 versus 229.5 ± 96.7, resp.; *P* < 0.01, both) (Figures 3(b) and 3(c)). By contrast, ALB levels on postoperative day 1 were significantly higher in subgroup C than subgroup D (33.1 ± 6.7 versus 27.2 ± 6.1, *P* < 0.01) (Figure 3(d)). In addition, the main postoperative complications were statistically less in subgroup C than subgroup D (63/235 versus 79/206, *P* < 0.01). No significant differences were observed in the remaining postoperative data.

In the prognosis analysis, recurrence time was significantly longer in subgroup C (235 patients) than subgroup D (206 patients) (7.1 ± 3.2 versus 4.9 ± 2.6, *P* < 0.01) (Figure 3(e)). Log-rank tests indicated that survival until the 24th month also differed significantly between subgroup C and subgroup D (Figure 3(f)). Based on the significant difference in survival rates in group 2, Cox regression model analysis was performed using the following multiple variables: age; gender; Child-Pugh grade; treatment; preoperative TBIL; preoperative AFP; preoperative viral load; surgical resection; and number of nodules. All the variables were selected by considering the possibility items of preoperative general information. The results of the Cox regression model analysis of group 2 indicated that treatments with SAMe (*P* = 0.013), Child-Pugh grade (*P* = 0.015), and viral load (*P* < 0.01) were independent variables that increased the survival rate (Table 3).

## 4. Discussion

SAMe supplementation restores hepatic GSH deposits and attenuates liver injury [11]. However, homeostasis of SAMe also regulates methionine adenosyltransferase activities and controls liver growth and cell apoptosis [21]. SAMe has been used for the treatment of chronic liver disease for decades. Our previous meta-analysis confirmed the protective effects and safety of SAMe for chronic liver disease [22]. Animal experiments have indicated that intrahepatic SAMe depletion is associated with hepatic fibrosis [23]. However, studies of the viral response have indicated that SAMe is correlated with an early viral response in chronic hepatitis C (CHC) patients [24, 25]. For CHB patients, the potential antiviral effect of SAMe was also demonstrated in hepatic cancer cell mechanism studies [19, 26]. However, the clinical efficacy of SAMe for HBV-related HCC has not been characterized. To improve our understanding of the clinical value of SAMe for HBV-related HCC with BCLC stages, we performed this research involving 697 patients.

SAMe exhibited protective effects for surgical resection of advanced-stage hepatocellular carcinoma (BCLC stages* B~C*) during the perioperative period. SAMe reduced postoperative ALT and AST levels and maintained postoperative ALB levels. Moreover, SAMe therapy reduced postoperative complications. However, for patients with early-stage hepatocellular carcinoma (BCLC stages* A1~A4*), SAMe therapy had limited clinical value. No benefit of SAMe was observed for small-size hepatocellular carcinoma during the perioperative period, except for maintaining ALB levels on postoperative day 1 (Figure 2). In addition, no significant clinical effects of SAMe therapy on long-term prognosis analysis were observed in group 1. By contrast, in group 2, long-term SAMe therapy significantly ameliorated prognosis.

These results can be summarized as follows. First, long-term administration of SAMe was also effective for HBV-related HCC patients. Second, SAMe treatment had different clinical effects in patients with different BCLC stages; SAMe was more effective for patients with advanced-stage tumors. Third, for patients with advanced-stage tumors, SAMe extended the recurrence time and improved survival. In addition, we determined that Child-Pugh grade and preoperative viral load were independent variables affecting the survival rate in addition to treatment with SAMe. Accordingly, we can propose some hypotheses to explain these results. As previously described, SAMe supplementation attenuates liver injury and controls liver growth. In an animal model, SAMe ameliorates oxidative stress status and reduces protein degradation [27]. We observed that the ALB in both groups and ALT and AST in group 2 were significantly higher in the SAMe therapy subgroup only on operative day 1 but not significant in later days. In view of the aforementioned words, this phenomenon may suggest that SAMe could improve early postoperative antistress function. Patients with advanced-stage tumors may also be subject to greater potential liver injury during the perioperative period and delayed restoration of liver function. SAMe supplementation would have clinical value for these patients. What is more, for long-term SAMe treatment, moderate and persistent oral administration of SAMe had protective effects for extending recurrence time, thereby increasing survival. SAMe may induce these effects by enhancing the antiviral activity of entecavir and improving liver function. We understood that many published literatures showed that SAMe exerted chemopreventive effects against HCC. However, rare studies reported SAMe could delay HCC recurrence and improve survival. This finding meant that exogenous SAMe supplement was safe and could ameliorate liver function. More importantly, SAMe could be used as a regular treatment for prevention of HBV-related HCC and prolonged the disease-free survival time. In addition, Cox regression model analysis identified Child-Pugh grade and preoperative viral load as independent factors significantly associating with the survival rate. Patients with worse Child-Pugh grade are more likely to undergo hepatic surgery and have worse prognosis. Moreover, a higher viral load, as an independent variable, increases the susceptibility of patients to cirrhosis and can affect the survival rate, as demonstrated in a previous large-sample controlled study [28]. Nevertheless, the efficacy of SAMe should be further tested.

For the therapeutic strategies of BCLC stage system, the patients in advanced stages should be treated with TACE or sorafenib (BCLC stages* B~C*). And hepatic resections should only be used in patients with early stages (BCLC stages* 0~A*). However, in recent years, researches exhibited that hepatic resection could be used in patients with advanced stages and brought better outcomes [29–31]. Our research group started hepatic resections for patients with advanced stages HCC since the 1990s. Based on our previous medical cases, according to BCLC stages, we retrospectively collected 697 patients to perform this research. This research provides the first comprehensive statistical analysis of the efficacy of SAMe for the treatment of patients with hepatitis B-related HCC with tumors of different BCLC stages. However, despite our strict inclusion criteria, this research is subject to limitations. First of all, because individual differences and multiple factors were present, our conclusions may be modified by some unidentified influencing factors. Then, due to unknown confounding factors, our trial design and statistical methods may have been inappropriate for some analyses. Thirdly, the sample size (especially in group 1) may be insufficient and the conclusion may be not reliable enough. Lastly, as mentioned above, we neglected to perform blood viral titer test, although we speculated that SAMe could delay recurrence time in group 2 due to enhancing the antiviral activity of entecavir. The results of blood tests may exhibit clinical significance and this should be conducted in the future randomized prospective clinical trials. Clinical trials are needed to address these shortcomings.

In conclusion, despite the limitations of this analysis, we demonstrated that SAMe is effective for HBV-related HCC patients. Importantly, SAMe exhibited significant clinical value for patients with advanced tumors (BCLC stages* B-C*) and improved prognosis. However, the clinical efficacy of SAMe for patients with early-stage tumors (BCLC stages* A1–A4*) was limited. The efficacy of SAMe therapy should be also explored further in randomized prospective clinical trials.

## Figures and Tables

**Table 1:** General information for the included patients at entry.

	BCLC stages A1–A4 (*n* = 256)			BCLC stages B-C (*n* = 441)		
	MgiBC + SAMe	MgiBC	*P* value	MgiBC + SAMe	MgiBC	*P* value
Gender (M/F)	83/36	106/31	0.16	192/43	177/29	0.23
Age (years)	55.6 ± 6.1	56.3 ± 6.9	0.39	58.1 ± 8.4	57.6 ± 9.3	0.81
Liver function						
TBIL (umol/L)	23.2 ± 9.7	24.3 ± 10.6	0.38	30.1 ± 11.5	28.1 ± 11.9	0.07
ALT (U/L)	36.6 ± 12.1	35.9 ± 13.3	0.66	41.3 ± 13.3	43.2 ± 15.1	0.16
AST (U/L)	46.7 ± 14.9	44.3 ± 14.1	0.18	49.1 ± 13.7	47.9 ± 12.6	0.34
ALB (g/L)	34.9 ± 5.1	35.9 ± 6.1	0.15	32.6 ± 7.7	33.2 ± 8.3	0.43
Lesion data						
Nodules (1/≥2)	106/13	120/17	0.71	211/24	189/17	0.47
Size (cm)	3.4 ± 1.1	3.6 ± 1.2	0.16	7.4 ± 3.6	7.9 ± 3.2	0.12
Tumor staging (*n*)						
A1~A3	106	120	0.71	—	—	—
A4	13	17		—	—	
B	—	—	—	190	167	0.95
C	—	—		45	39	
Child-Pugh^*∗*^						
Class A/B (*n*)	82/37	103/34	0.26	183/52	165/41	0.56
AFP (ng/mL)	776.1 ± 349.1	801.7 ± 364.8	0.56	917.2 ± 425.7	868.1 ± 410.5	0.21
Viral load (UI/uL)	401.6 ± 203.6	381.9 ± 187.3	0.42	429.3 ± 236.7	447.1 ± 263.5	0.45

^*∗*^Child-Pugh class B patients who received symptomatic treatment were considered Child-Pugh class A prior to surgery.

**Table 2:** Clinical characteristics of the included patients after surgery.

Items	BCLC stages A1–A4 (*n* = 256)			BCLC stages B-C (*n* = 441)		
	MgiBC + SAMe	MgiBC	*P* value	MgiBC + SAMe	MgiBC	*P* value
Postoperative ICU stay (days)	1.2 ± 0.2	1.2 ± 0.4	1.00	1.2 ± 0.1	1.2 ± 0.2	1.00
Patients with postoperative complications (*n*)	36	43	0.84	63	79	<0.01
Biliary fistula	5	6	0.94	21	21^◆^	0.65
Poor wound healing	12	15^▲^	0.86	21^●^	19	0.91
Wound infection	8	6	0.41	7	13^■^	0.09
Abdominal infection	2	5	0.33	3	3	0.87
Intestinal obstruction	0	1	0.35	3	5	0.36
Pulmonary infection	4	3	0.56	2	5	0.18
Peritoneal effusion	3	5^▲^	0.60	6^●^	11^◆^	0.12
Subphrenic abscess	2	3	0.76	3	7^■^	0.13
*Surgical resection*						
Limited resection	81	103	0.20	153	133	0.90
Left lateral segmentectomy	14	11	0.31	29	25	0.94
Left hemihepatectomy	11	17	0.41	35	37	0.38
Extended left hemihepatectomy	2	1	0.48	5	2	0.33
Right hemihepatectomy	11	5	0.06	12	9	0.71
Extended right hemihepatectomy	0	0	—	1	0	0.34
*Surgical data*						
Surgical time (min)	196.4 ± 63.1	187.4 ± 59.3	0.24	231.5 ± 112.9	225.7 ± 105.9	0.57
Surgical hemorrhage (mL)	232.4 ± 99.4	219.9 ± 110.1	0.34	348.7 ± 148.8	366.3 ± 153.2	0.22
Postsurgical hospital stay (days)	16.7 ± 5.7	17.5 ± 5.3	0.24	20.6 ± 7.1	21.0 ± 6.9	0.55

^▲^One patient in group 1 with postoperative poor wound healing also exhibited peritoneal effusion. ^●^Three patient in group 2 with postoperative poor wound healing also exhibited peritoneal effusion. ^◆^Two patients in group 2 with biliary fistulae exhibited peritoneal effusion. ^■^Three patients in group 2 with subphrenic abscess exhibited wound infection.

**Table 3:** Results of Cox regression model analysis of patient survival in group 2.

Variable	Hazard ratio	95% CI		*P* value
		Lower	Upper	
Age	0.983	0.960	1.006	0.150
Gender	1.067	0.757	1.503	0.711
SAMe treatment	0.736	0.578	0.938	0.013
Preoperative AFP	1.000	0.999	1.001	0.203
Preoperative TBIL	0.807	0.515	1.264	0.349
Surgical resection	0.969	0.873	1.075	0.550
Nodule number	0.834	0.581	1.196	0.323
Preoperative Child-Pugh grade	0.697	0.521	0.932	0.015
Preoperative virus load	1.001	1.000	1.001	0.000
